# The Potential Role of Glucagon-Like Peptide-1 (GLP-1) Receptor Agonists and Glucose-Dependent Insulinotropic Polypeptide (GIP) Receptor Agonists in Obstructive Sleep Apnea and Obesity

**DOI:** 10.1007/s13665-025-00384-1

**Published:** 2025-07-25

**Authors:** Charlotte C. Ellberg, Hannah Schwartz, Annika Witt, Karen C. McCowen, Ana Lucia Fuentes, Atul Malhotra

**Affiliations:** 1 Department of Medicine, University of California, San Diego, La Jolla, CA USA; 2 Department of Medicine, Weill Cornell Medicine, New York, NY USA; 3 Brown University, Providence, RI USA; 4 Division of Endocrinology, University of California, San Diego, La Jolla, CA USA; 5 Division of Pulmonary, Critical Care, Sleep Medicine and Physiology, University of California, Gilman Drive, San Diego, La Jolla, CA 9500 USA

**Keywords:** Cardiometabolic, GLP-1 receptor agonist, GIP receptor agonist, Weight loss, Obesity, lung

## Abstract

**Purpose of Review:**

Strong associations exist between obstructive sleep apnea (OSA) and obesity. Prior studies have demonstrated that weight reduction in people with OSA and obesity improves severity of OSA. Until recently, there were no approved pharmacotherapies for OSA. We aim to review recent literature on GLP-1 receptor agonists and GIP agonists and their potential role in the management of OSA.

**Recent Findings:**

Novel pharmacotherapies developed to target obesity include glucagon-like peptide-1 (GLP-1) receptor agonists and glucose-dependent insulinotropic polypeptide (GIP) receptor agonists. These therapies have proven to be helpful in many comorbid conditions, with published studies suggesting a benefit in OSA.

**Summary:**

GLP-1 receptor agonists and GIP agonists are emerging potential therapies for OSA and associated cardiometabolic risk.

## Introduction

Obstructive sleep apnea (OSA) is a form of sleep-disordered breathing associated with several poor outcomes including depression, impaired memory, and hypertension [1–4]. OSA increases the risk of motor vehicle accidents, and may also increase the risk of myocardial infarction and stroke [5, 6]. Recent estimates suggest that OSA affects up to 1 billion people worldwide [7]. Of note, this number is growing over time due to the obesity pandemic, the aging of the population, and increasing awareness of the importance of diagnosing OSA [8]. Risk factors for OSA include obesity, male sex, age, and genetic factors [9]. Current management of OSA includes continuous positive airway pressure (PAP) [10], oral appliances, and weight reduction strategies [11, 12]. Because obesity is one of the strongest risk factors for OSA, a focus on weight reduction has emerged as an effective management strategy. Studies have demonstrated treatment of obesity and OSA with bariatric surgery improves OSA outcomes [13]. Emerging studies are highlighting the role of pharmacotherapies for weight loss and OSA [14].

### Current Therapies for OSA

Nasal PAP has been shown in randomized trials to improve daytime symptoms and reduce systemic blood pressure [18–20]. For some patients, nasal PAP can have transformative benefits [18]. Recent data suggest adherence rates of up to 87% based on US Medicare criteria, especially when using modern tools such as patient engagement technologies [19]. However, PAP adherence remains variable, prompting ongoing efforts to seek alternative therapies. In addition, PAP therapy has been shown consistently to lead to small but significant increases in body weight, although mechanisms for this observation remain unclear [20]. Oral appliances and upper airway surgery have also been studied as treatment options for OSA, but outcomes are mixed, and predicting good candidates remains a challenge [24–28]. Thus, these treatments are considered second line if PAP therapy fails. Although a recent meta-analysis showed some benefits in all-cause mortality and cardiovascular mortality [10], PAP has not been shown in randomized trials definitively to improve hard cardiovascular outcomes [26], highlighting the need to improve adherence to therapy and to address underlying causes of OSA such as obesity. Thus, considerable focus has been placed on the need to treat both OSA as well as obesity rather than either condition alone [27].

### The Role of Weight Loss in OSA

In addition to nasal PAP, weight loss has been identified as a targeted intervention for OSA. Recently, a meta-analysis highlighted various weight loss strategies including bariatric surgery, pharmacotherapy and lifestyle interventions (diet and exercise) [12]. In aggregate, the data showed a greater degree of sleep apnea improvement (as judged by the apnea hypopnea index [AHI]) with greater weight reduction, regardless of how it was achieved. The bariatric surgery groups had the greatest weight loss resulting in large improvements in AHI, as compared to pharmacotherapy and lifestyle interventions. The analyses led to some debate regarding the effect of weight loss on AHI. Some argue that the effect is linear i.e. for every pound lost there is a corresponding improvement in AHI, while others suggest a curvilinear or quadratic function or threshold whereby a certain amount of weight loss leads to marked improvement or even resolution of OSA. This meta-analysis predated the SURMOUNT-OSA results but supported the notion that weight loss is beneficial for OSA treatment [12].

In addition to evaluating the effects of weight loss on AHI, studies have also evaluated cardiovascular outcomes with respect to weight loss and OSA management. In one study of 181 patients with obesity, moderate to severe OSA, and elevated high sensitivity C-reactive protein (hsCRP) levels (> 1.0 mg/L), participants were randomized to receive PAP, weight loss intervention, or a combination of both over 24 weeks [27]. Outcomes assessed included hsCRP, markers of insulin sensitivity, lipid profiles, and blood pressure. In the weight loss-only and combination groups, there were significant reductions in hsCRP levels, and serum triglyceride levels along with improved insulin sensitivity; however, these reductions were not observed in the PAP-only group. All treatment groups showed reductions in blood pressure, though the combination arm had a greater decrease in both systolic blood pressure and mean arterial pressure compared to the other intervention groups. In aggregate, these data support the notion that treating both OSA and obesity has greater cardiometabolic benefits than treating either one alone.

Building on these findings, a subsequent study evaluated the combined effects of PAP and weight loss on cardiovascular risk using physiologic measures [28]. Adults with obesity and moderate-to-severe OSA were randomized to either PAP alone, weight loss facilitated by weekly counseling and progressive exercise (WL), or a combination of both over the course of 24 weeks. Central blood pressure and carotid-femoral pulse wave velocity (CF-PWV) were measured as indicators of cardiovascular risk. The combination group experienced a significant reduction of central systolic and diastolic blood pressure, while the other groups did not. No treatment group showed significant changes in central pulse pressure, pulse pressure amplification, the central augmentation index, or CF-PWV (after adjustments for mean arterial pressure and heart rate). Collectively, these studies highlight that in patients with obesity and OSA, combining PAP with weight loss yields greater improvement in cardiovascular risk profiles, particularly in reducing systolic blood pressure, than either treatment alone.

### Bariatric Surgery and OSA

Bariatric surgery has been studied as a therapy for weight loss and OSA. Meta-analyses have consistently highlighted reductions in both body mass index (BMI) and AHI with bariatric surgery [32–34]. One meta-analysis of 12 studies involving 342 patients found reductions in both BMI and AHI post-surgery [30]. However, among those with moderate-severe OSA, the mean AHI was statistically unchanged, underscoring the need for continued treatment of OSA beyond bariatric surgery. A more recent systematic review and meta-analysis published by Wong et al. [29] further supported these conclusions, reporting reductions in BMI and AHI after surgical weight loss, but noting persistent OSA at follow up [32, 33]. There are limited randomized controlled trials evaluating bariatric surgery for OSA. In one randomized trial comparing PAP to laparoscopic gastric banding in patients with severe OSA, PAP was associated with a lower effective AHI at 9 months, but there were no differences in daytime sleepiness between groups [34, 35]. In aggregate the data do support achieving weight loss in people with OSA [33], but resolution of OSA occurs infrequently without PAP therapy being given concomitantly.

### Physiology of Incretins

While weight loss has been shown to be beneficial for OSA, lifestyle modifications can be difficult to sustain, and bariatric surgery is not without risks, cost, and patient hesitancy [36]. For these reasons, studies have evaluated pharmacotherapies such as incretin-mimetics for obesity and OSA treatment. The main incretins relevant to this discussion are glucagon-like peptide 1 (GLP-1) and glucose-dependent insulinotropic polypeptide (GIP), and they are released by the entero-endocrine L cells in the intestine in response to the presence of nutrients in the gut lumen [37]. These hormones promote insulin production, and GLP-1 decreases glucagon secretion, suppresses appetite and slows gastric emptying. In addition, GLP-1 receptors are found not only in the pancreas, but also in the heart, stomach, adipose tissue, vague nerve, and central nervous system. GLP-1 receptor agonists (GLP1RA) developed for clinical use include liraglutide, semaglutide, exenatide, dulaglutide, lixisenatide, and abliglutide. Tirzepatide is the first dual GIP and GLP1RA developed which is now in widespread use.

GLP-1 receptors, widespread in the brain and particularly in areas of the mesolimbic reward pathway, also play a role in reward signaling. Rigorous studies performed in rats have shown a connection between the incretins and dopamine signaling, while the human connection is still uncertain [38]. In the lateral septum, a brain region that is partly associated with reward, GLP-1 receptors are plentiful along with dopamine receptors [39]. GLP-1 has been shown to increase dopamine uptake and clearance along with increasing dopamine transporter expression in the striatum. As a result of these effects on the dopamine reward pathways, some have speculated the GLP1RA can impact hedonic feeding i.e. eating for pleasure and reward rather than for hunger or physiological need. GLP1RA also exhibits effects on orexin which may be important for sleep/wake regulation [40]. Given the widespread nature of the GLP1 receptors throughout the brain [41], further work is clearly needed regarding mechanisms of action of various pharmacotherapies. The central effects of GIP are also of substantial interest, but less well defined [42].

### GLP1 Receptor Agonists and Weight Loss

GLP1RAs have been available for about 20 years. They were initially approved for type 2 diabetes mellitus (DM) based on improvements in glycemic control, along with reductions in body weight. More recently tirzepatide (dual agonist) was approved for DM [43], then for obesity and most recently for OSA.

Jastreboff et al. have published a number of prominent studies regarding tirzepatide therapy for obesity [44, 45]. In a major randomized, placebo-controlled trial, the authors demonstrated > 20% weight loss with tirzepatide treatment at 72 weeks [44]. The authors recently reported three-year follow-up showing a marked reduction in incident diabetes [45]. These data suggest major improvements in metabolic health with tirzepatide therapy that are sustainable over time.

### GLP1 Receptor Agonists, Weight Loss, and OSA

Given the effects of GLP1RA on weight loss, these pharmacotherapies have been examined as a potential treatment for OSA. Earlier studies evaluated liraglutide, a GLP-1RA approved for weight loss and diabetes. In the SCALE Sleep Apnea randomized clinical trial, liraglutide was associated with significantly greater reductions in AHI, body weight, systolic blood pressure, and HbA1c when compared to placebo among participants with obesity and moderate to severe OSA [46]. A meta-analysis including this trial and five additional studies evaluated GLP-1RA in OSA with AHI outcomes [47]. Among a total of 1067 participants, GLP-1RA (particularly dual agonists with GIP activity) significantly decreased AHI, promoted weight loss, and lowered blood pressure [48, 49].

Tirzepatide, a dual agonist, has shown even greater efficacy in weight loss than GLP1RA alone [44, 45, 50].Tirzepatide was recently FDA-approved as the first pharmacotherapy for OSA. The SURMOUNT-OSA trials were two phase 3 randomized, placebo-controlled trials to evaluate the safety and efficacy of tirzepatide for managing moderate to severe OSA in participants with obesity [51, 52]. Trial one included those not utilizing PAP therapy, and trial 2 included participants utilizing PAP therapy. 469 participants with moderate to severe OSA were randomized to receive either maximally tolerated dose of tirzepatide or placebo for 52 weeks. Among patients with OSA in both trials (with and without PAP), tirzepatide yielded reduced AHI, body weight, sleep apnea specific hypoxic burden, hsCRP concentrations, systolic blood pressure, and improved patient reported outcomes (Table 1).

**Table 1:** Summary of studies on GLP-1RA and OSA outcomes

Study	Therapy	Sample Size	Study Design	Key Findings
Malhotra et al. [52] SURMOUNT-OSA	Tirzepatide	469 patients with OSA and obesity	Phase 3 double-blind randomized controlled trial	Tirzepatide reduced AHI, body weight, hypoxic burden, hsCRP concentration, systolic blood pressure, and improved sleep-related, patient reported outcomes
Gomez-Peralta et al. [68]	Liraglutide	158 obese patients with T2DM and OSA	Single-center, retrospective study	Reduction in ESS scores at one and three months with liraglutide
Jiang et al.[69]	Liraglutide plus CPAP vs. CPAP alone	90 Patients with T2DM and severe OSA	Two-center, prospective randomized controlled trial	Reduced BMI, systolic blood pressure, AHI scores, and hypoxia in combination group
Blackman et al. SCALE Sleep Apnea randomized clinical trial [46]	Liraglutide vs. placebo	276 non-diabetic obese patients with OSA	Randomized, double-blind trial	Greater reduction in mean AHI, body weight, systolic blood pressure, and HbA1c in liraglutide group compared to placebo

The SURMOUNT-OSA study had compelling results for the treatment of moderate-to-severe sleep apnea in people with obesity. However, a number of points are important regarding the generalizability of these findings:

- SURMOUNT-OSA does not pertain to all patients with OSA. For example, patients with mild OSA (AHI of 5–15/hr), people with DM, people who were lean (BMI < 25 kg/m2) or overweight (BMI 25–30 kg/m^2^) were not included in the study and thus conclusions are limited to the population studied [53].
- The trial had one year of follow-up and thus long-term conclusions regarding hard cardiovascular outcomes are limited. Of note, weight loss is ongoing at one year in the study and the side effects of the tirzepatide attenuate over time, thus the long-term continuation of therapy may well be viable. Thus, the data are suggestive but not definitive that ongoing tirzepatide therapy should help hard cardiovascular outcomes in patients with OSA. Discontinuation of tirzepatide therapy would be predicted to yield weight regain based on the SURMOUNT 4 study recently published in JAMA [54].
- The SURMOUNT-OSA study was placebo controlled but did not compare active treatment as would be undertaken in comparative effectiveness research [55]. Future studies should address whether PAP therapy, tirzepatide therapy, or a combination are better or worse than either alone. Based on prior reports, the combination of treating both OSA and body weight is likely better than treating either condition alone.

As with any novel treatment, questions remain both for clinicians and for researchers.

For the clinician:

- Can diet and exercise be implemented sufficiently to maintain weight loss once tirzepatide therapy has been given, or is long term pharmacotherapy required? The SURMOUNT 3 study reported benefits to tirzepatide following intensive diet/exercise^56^ but the approach of maintaining weight loss behaviorally after pharmacotherapy is less well studied. Clinical experience suggests that some patients can behaviorally sustain weight loss when it was initially achieved pharmacologically.
- Could tirzepatide therapy be used adjunctively if alternative therapies are partially successful? For example, many patients who receive upper airway surgery or mandibular advancement therapy get improvement but not resolution of their OSA [31, 57]; could tirzepatide be used to eliminate OSA in such patients?
- Although complex medical patients were excluded from SURMOUNT-OSA, should tirzepatide be used in patients with OSA plus DM or congestive heart failure [58], based on previously published studies in patients with these comorbidities? (see Table 2).

For the researchers, a number of questions remain as well:

- Can oral agents such as orforglipron (a novel nonpeptide GLP1-RA, not yet FDA approved) be used to maintain weight loss following tirzepatide induced weight loss [59],? Ongoing studies are addressing this question. Other agents such a triple-hormone receptor agonist retatrutide (agonist of GLP1-RA, GIP and glucagon) are also being investigated [60].
- Can long term studies be undertaken to determine if the observed results in SURMOUNT-OSA are sustained over time? Such studies could be used to assess the impact of treatment on hard cardiovascular outcomes such as myocardial infarction and stroke. Cost effectiveness studies are needed given the expense of these medications. Comparative effectiveness studies should assess differences between active treatments such as bariatric surgery vs. tirzepatide therapy in OSA. Patient preferences are also critical to assess in this context.
- Mechanistic studies are also important to consider, as weight loss alone may explain some but not all of the observed improvements with tirzepatide therapy. GLP1RAs have activity at the carotid body, in the hypothalamus, and other central nervous system areas suggesting that some of the observed improvements may be complex e.g. reductions in sympathetic activity may occur via the carotid body and could mediate some of the observed improvements in blood pressure [61]. Mediation analyses are now being conducted to determine the impact of OSA improvement vs. obesity improvement vs. direct effects of the medications on outcomes of interest in SURMOUNT-OSA as well.
- One concern with major weight loss is the potential for sarcopenia related to loss of skeletal muscle [62]. Of note, basal metabolic rate is a function of skeletal muscle mass. Thus the ability to maintain weight loss may be impaired if skeletal muscle mass is not preserved. Strength training exercise and protein intake has been suggested as interventions which could be used to preserve skeletal muscle mass in the context of weight loss studies [63]. Pharmacotherapies such as bimagrumab (a human monoclonal antibody that inhibits activin receptors and promotes muscle hypertrophy) are also being considered to address skeletal muscle function in the context of weight loss [64]. Androgen modulators are also being actively investigated in this context [65].

**Table 2:** Table outlining current strategies for OSA management

Treatment	Mechanism of action	Effect on OSA	Considerations
CPAP	Positive airway pressure to maintain patent airways	Improves AHI, reduces daytime sleepiness	Requires compliance, may not address underlying obesity or hard cardiovascular outcomes
GLP-1 receptor agonists	Appetite suppression, insulin sensitivity, weight loss	Reduces AHI through weight loss and possibly airway function	High cost, requires self injection, gastrointestinal side effects
GLP1/ GIP agonist (i.e. tirzepatide)	Dual GLP-1/GIP for weight loss and improved insulin sensitivity	Emerging evidence suggests improvement in OSA severity	High cost Requires self injection, risk of gastrointestinal side effects
Phentermine/topiramate	Appetite suppression	Some improvement	Not FDA approved for sleep apnea; modest effects^70^
Behavioral weight loss strategies	Calorie deficits leading to fat loss in neck area	Reduction in OSA severity with sufficient weight loss	Requires lifestyle commitment
Bariatric surgery	Reduction of stomach size to induce weight loss; some procedures malabsorptive in nature	Long-term reduction in OSA severity in obese patients	High cost, surgical risks and recovery from surgery, patient hesitancy

## Conclusions

Considerable progress has been made with regard to drug therapy for OSA [1, 14, 66, 67]. Tirzepatide represents a major first step towards OSA pharmacotherapy, primarily targeting weight loss and cardiometabolic risk. Ongoing research is encouraged to address the major global burden of disease.

## Key References

• Of Importance:

- 1. Jordan AS, McSharry DG, Malhotra A. Adult obstructive sleep apnoea. Lancet. Feb 22 2014;383(9918):736-47. doi:10.1016/S0140-6736(13)60734-5
  - This review summarizes the start of the art on sleep apnea pathogenesis inclduing the rationale for individualized treatment.
- 53. Wharton S, Blevins T, Connery L, et al. Daily Oral GLP-1 Receptor Agonist Orforglipron for Adults with Obesity. N Engl J Med. Sep 7 2023;389(10):877-888. doi:10.1056/NEJMoa2302392
  - This study is the first major study of orforglipron to treat obesity.

•• Of major Importance:

- ▪▪.10 Benjafield AV, Ayas NT, Eastwood PR, et al. Estimation of the global prevalence and burden of obstructive sleep apnoea: a literature-based analysis. Lancet Respir Med. Aug 2019;7(8):687-698. doi:10.1016/S2213-2600(19)30198-5.
  - This paper is heavily cited as the best available estimate of the global prevalence of obstructive sleep apnea.
- Benjafield A, Pepin, JL, Cistulli, PA, Wimms, A, Lavergne, F, Kuniyoshi, FHS, Munson, SH, Schuler, B, Badikol, SR, Wolfe, KC, Willes, L, Kelly, C, Kendzerska, T, Johnson, DA, Heinzer, R, Lee, C-H, Malhotra, A, on behalf of the medXCloud group. Positive airway pressure therapy and all-cause and cardiovascular mortality in people with obstructive sleep apnoea: a systematic review and meta-analysis of randomized clinical trials and confounder-adjusted, non-randomised controlled studies. Lancet Respiratory Medicine. 2025, 13(5): 403–413.
  - This study is a recently published meta-analysis of randomized and counfounder-adjusted non-randomized studied for CPAP for treating sleep apnea.
- ▪▪ 47. Malhotra A, Grunstein RR, Fietze I, et al. Tirzepatide for the Treatment of Obstructive Sleep Apnea and Obesity. N Engl J Med. Jun 21 2024;doi:10.1056/NEJMoa2404881
  - This publication led to FDA approval of tirzepatide for treatment of obstructive sleep apnea. It represents two major multicenter placebo controlled randomized trials.

## Data Availability

No datasets were generated or analysed during the current study.
